# Supplementary material for: Content-rich biological network constructed by mining PubMed abstracts
Source: BMC Bioinformatics. 2004 Oct 8;5:147. doi: 10.1186/1471-2105-5-147 (PMC528731; doi:10.1186/1471-2105-5-147)
Supplement: Additional File 5 — The original Chilibot query results of the term "long-term potentiation (LTP)" and 22 other terms, limiting the latest references analyzed to the years 1990, 1995, 2000, and 2004. [file 1471-2105-5-147-S5.bz2 › chilibotAdditionalFile5/ltp2004/html/TAU_KV4.2.html]

 


 **TAU** and **KV4.2** 
  
Found 5 abstracts in PubMed,  **5 abstracts were retrieved and analyzed**.  


---

 Search Google  |
 PDF files only 
|  EDU domain only 

---

**Interactive relationship** (e.g. stimulation, inhibition, etc)

- The slow component was moderately accelerated by the truncation  **tau**  = 346 ms and accounted for 11 % of the total  **Kv4.2**  current inactivation.  Ref: 11507158 J Physiol, 2001

- :-)

  **Parallel relationship** (e.g. studied together, co-existance, homology, etc.)

  - Both the fast and the intermediate components of inactivation were slowed by a deletion of the  **Kv4.2**  N terminus  **tau**  = 35 and 111 ms and accounted for 33 and 56 %, respectively, of the total decay.  Ref: 11507158 J Physiol, 2001
  - The time constants  **tau**  of recovery from inactivation of I A and  **Kv4.2**  were 27.9 14.1 ms and 172.8 10.0 ms, respectively.  Ref: 14695490 Sheng Li Xue Bao, 2003
